# Supplementary material for: A whole-animal phenotypic drug screen identifies suppressors of atherogenic lipoproteins
Source: eLife. 2026 Aug 27;14:RP105314. doi: 10.7554/eLife.105314 (PMC13521566; doi:10.7554/eLife.105314)
Supplement: Supplementary file 2 [file elife-105314-code1.zip › JHDL_primary_result/TRIPTONIDE/TRIPTONIDE_JHDL_7_035_005_2019_06_14_11_14.pdf]

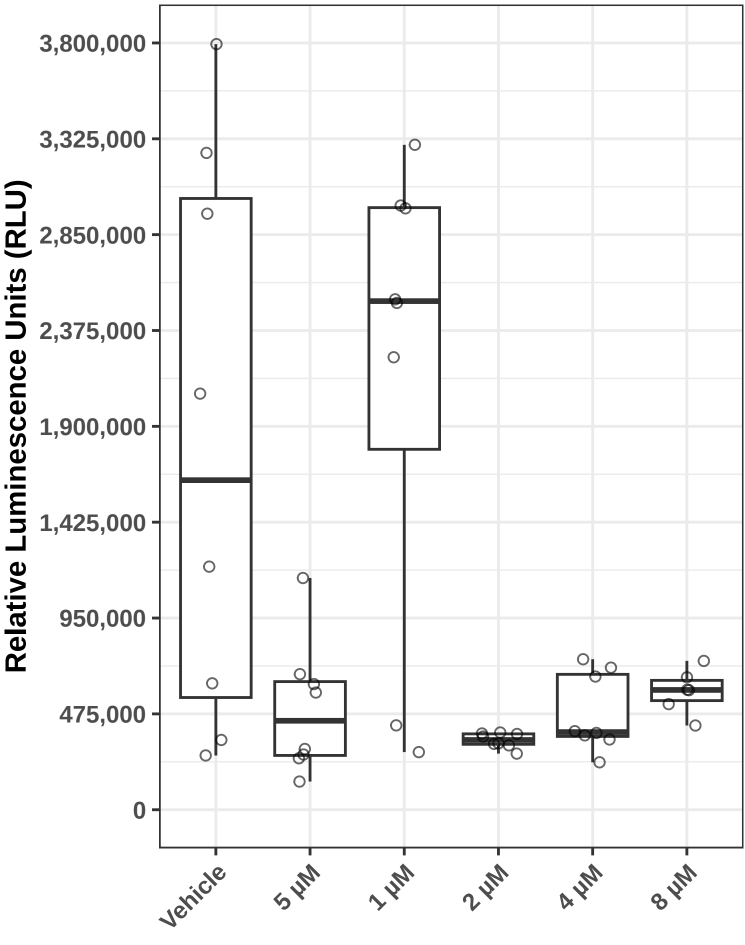
Relative Luminescence Units (RLU)
3,800,000
3,325,000
2,850,000
2,375,000
1,900,000
1,425,000
950,000
475,000
0
Vehicle
5 µM
1 µM
2 µM
4 µM
8 µM
